# Analysis of Scientific Publications During the Early Phase of the COVID-19 Pandemic: Topic Modeling Study

**DOI:** 10.2196/21559

**Published:** 2020-11-10

**Authors:** Andreas Älgå, Oskar Eriksson, Martin Nordberg

**Affiliations:** 1 Department of Clinical Science and Education, Södersjukhuset Karolinska Institutet Stockholm Sweden; 2 Department of Global Public Health Karolinska Institutet Stockholm Sweden; 3 DataRobot Inc Stockholm Sweden

**Keywords:** COVID-19, SARS-CoV-2, coronavirus, pandemic, topic modeling, research, literature

## Abstract

**Background:**

The COVID-19 pandemic has spread at an alarming speed, and effective treatment for the disease is still lacking. The body of evidence on COVID-19 has been increasing at an impressive pace, creating the need for a method to rapidly assess the current knowledge and identify key information. Gold standard methods such as systematic reviews and meta-analyses are regarded unsuitable because they have a narrow scope and are very time consuming.

**Objective:**

This study aimed to explore the published scientific literature on COVID-19 and map the research evolution during the early phase of the COVID-19 pandemic.

**Methods:**

We performed a PubMed search to analyze the titles, keywords, and abstracts of published papers on COVID-19. We used latent Dirichlet allocation modeling to extract topics and conducted a trend analysis to understand the temporal changes in research for each topic, journal impact factor (JIF), and geographic origin.

**Results:**

Based on our search, we identified 16,670 relevant articles dated between February 14, 2020, and June 1, 2020. Of these, 6 articles were reports from peer-reviewed randomized trials on patients with COVID-19. We identified 14 main research topics, of which the most common topics were health care responses (2812/16,670, 16.86%) and clinical manifestations (1828/16,670, 10.91%). We found an increasing trend for research on clinical manifestations and protective measures and a decreasing trend for research on disease transmission, epidemiology, health care response, and radiology. Publications on protective measures, immunology, and clinical manifestations were associated with the highest JIF. The overall median JIF was 3.7 (IQR 2.6-5.9), and we found that the JIF for these publications declined over time. The top countries producing research were the United States, China, Italy, and the United Kingdom.

**Conclusions:**

In less than 6 months since the novel coronavirus was first detected, a remarkably high number of research articles on COVID-19 have been published. Here, we discuss and present the temporal changes in the available COVID-19 research during the early phase of the pandemic. Our findings may aid researchers and policy makers to form a structured view of the current COVID-19 evidence base and provide further research directions.

## Introduction

The novel coronavirus (SARS-CoV-2) was first detected in the Hubei Province in China in December 2019 [1]. The virus is known to cause a severe respiratory disease (COVID-19) that has rapidly spread worldwide [2]. On March 11, 2020, the World Health Organization declared the COVID-19 outbreak a pandemic [3]. Owing to the novelty of the disease, its clinical course and treatment are largely unknown [4]. However, the scientific society has mobilized quickly, and by June 1, 2020, more than 1300 clinical trials had been registered at various clinical trial registry sites [5,6].

Keeping track of the growing evidence base in medicine is becoming increasingly difficult owing to a large number of publications [7]. A rapid assessment of a dynamic research field such as COVID-19, where the body of evidence has been increasing at an impressive pace, requires an approach that is more direct and has a wider scope than that of the current gold standard methods, such as scoping and systematic reviews [8]. The potential uses of machine learning and artificial intelligence in the fight against the COVID-19 crisis has been previously discussed [9].

Several systematic reviews have been published on specific aspects of the pandemic, such as the impact of comorbidities, symptoms, and treatments [10-12]. However, a comprehensive assessment of all the available scientific publications on COVID-19 is lacking. Therefore, we aimed to explore the published scientific literature on COVID-19, assess relevant topics, and map the research evolution during the early phase of the COVID-19 pandemic by using a machine learning–based approach.

## Methods

### Data Collection

We searched PubMed on June 1, 2020, using PubMed E-utilities [13] and the search terms “covid[Title/Abstract] OR covid-19[Title/Abstract]”, with no language or date restrictions, utilizing the Biopython package for Python 3.6 (Python Software Foundation). The focus of this study was the disease (COVID-19) as opposed to the virus; hence, we refrained from including search terms such as “coronavirus”. We retrieved the title, keywords, abstract, date of last revision, list of author affiliation, journal name, and PubMed identifier number for each publication. We used the date of last revision as the indexing date for all articles, as this date was the readily available for all the data, unlike the date of submission or publishing.

### Data Preprocessing

**Figure 1:**
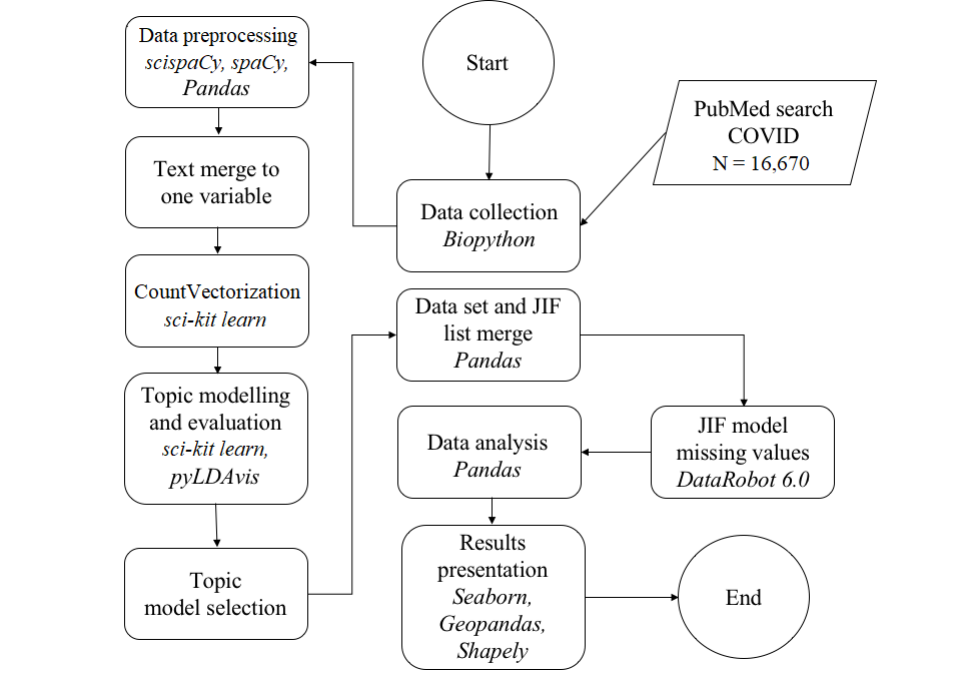
Data processing flowchart. Italicized text indicate the method used. JIF: journal impact factor.

All text data were lowercased and cleaned for double spaces, special characters, and numbers. Subsequently, we applied a list of stop words from PubMed [14], general English stop words, and subject-specific stop words (eg, covid, corona) to the titles and abstracts to remove non–information-bearing words from the text (Multimedia Appendix 1). The data processing workflow used is depicted in Figure 1.

We lemmatized the text data using the Python package scispaCy. We considered the country of the first author’s affiliation to be the country of origin and extracted geographic entities from the affiliations using the Python package spaCy. If a country name was not included in the affiliation, we used the last geographic entity mentioned and manually linked this geographic entity to a country; for instance, “New York” was linked to “the United States.”

To identify randomized clinical trials, we searched for the words “randomis*” and “randomiz*” in the titles. We then manually assessed all articles identified as potential randomized clinical trials to determine their true article type.

### Latent Dirichlet Allocation

Latent Dirichlet allocation (LDA) is a generative statistical model for data collection [15] that has previously shown to be suitable for topic modeling in medicine [16,17]. We used LDA to derive useful information from the identified articles. We concentrated all the textual data collected from each article (ie, title, keywords, and abstracts when available in PubMed) into one variable. We then used this variable as the text corpus for the whole data set and subsequently vectorized the variable using CountVectorizer in the Python package scikit-learn.

To assess the topic spaces in an interspersed arbitrary selection of topic numbers, we computed LDA models and principal component analysis (PCA) plots for 8, 13, and 35 topics. After assessing the results, we subsequently computed LDA models for all numbers of topics between 3 and 23. We chose the numbers 3 and 23 because fewer than 3 topics were considered too few for any fathomable use case and more than 23 topics were considered not useful or comprehensible, based on expert opinion. We recorded the evaluation metrics for perplexity, leave-out likelihood, and graphical PCA for each model. We decided the final number of topics based on the assessments of these three evaluation metrics, as well as the authors’ domain knowledge of COVID-19 and medical research.

Thereafter, 6 experienced clinicians and researchers independently labeled the identified topics based on the 15 most-common keywords for the articles assigned to each topic and the resulting most-frequent words in the LDA model for each topic (Multimedia Appendix 2). We then discussed the proposed labels until a consensus for each topic label was reached. We performed LDA modeling using the Python package scikit-learn and plotted the results using the package pyLDAvis [18].

We identified the most probable topic of each article and assigned it as the articles’ main topic. The weekly number of articles for each topic was then computed and the time series for the proportion of each topic during the week were plotted for further analysis.

### Journal Impact Factor

We looked up the journal names of all articles obtained from our PubMed search against the 2019 list of journal impact factor (JIF; Journal Citations Report, Clarivate Analytics), which covered 12,515 scientific journals [19]. Journal names were matched using DataRobot 6.0 (DataRobot Inc.) fuzzy matching, wherein a 90% similarity was considered a match. We then manually corrected the list of matched journal names to avoid mismatch. In cases where the journal name of the article could not be matched against the Clarivate JIF list, we developed a random forest regression model in DataRobot to predict the JIF from the article data.

DataRobot automatically performs a modelling competition in which a wide selection of algorithm and data preprocessing techniques compete with one another. The model with the best root mean square error (RMSE) and R^2^ on hold-out data is then chosen as the champion model. A modelling competition is beneficial because the same algorithm can have different efficiencies on different data sets. The article data that we could not match with the Clarivate JIF list originated from the journals that were not identified by the trained model. Therefore, we excluded all journal-specific variables when using the model, to avoid overfitting the model to the training data. To evaluate the model, we used a test sample and RMSE and R^2^ as model performance metrics. In the model, we used affiliations of the authors; keywords; first author’s affiliation; and all text in the title, keywords, and abstract—together and as separate variables. We built and deployed the random forest regression model in DataRobot.

Subsequently, we computed the median JIF for each identified topic. We also calculated the median JIF over time and the median JIF in correlation to the number of articles.

### Data Analysis

We performed all data analyses and visualizations using the Python packages Panda, NumPy, Matplotlib, pyLDAvis, and Seaborn (Python Software Foundation).

## Results

### Search Results

We found 16,670 articles dated between February 14, 2020, and June 1, 2020. Of these 16,670 articles, abstracts were available for 8560 (51.34%) articles, whereas title and keywords were available for 16,623 (99.71%) and 7739 (46.42%) articles, respectively.

**Figure 2:**
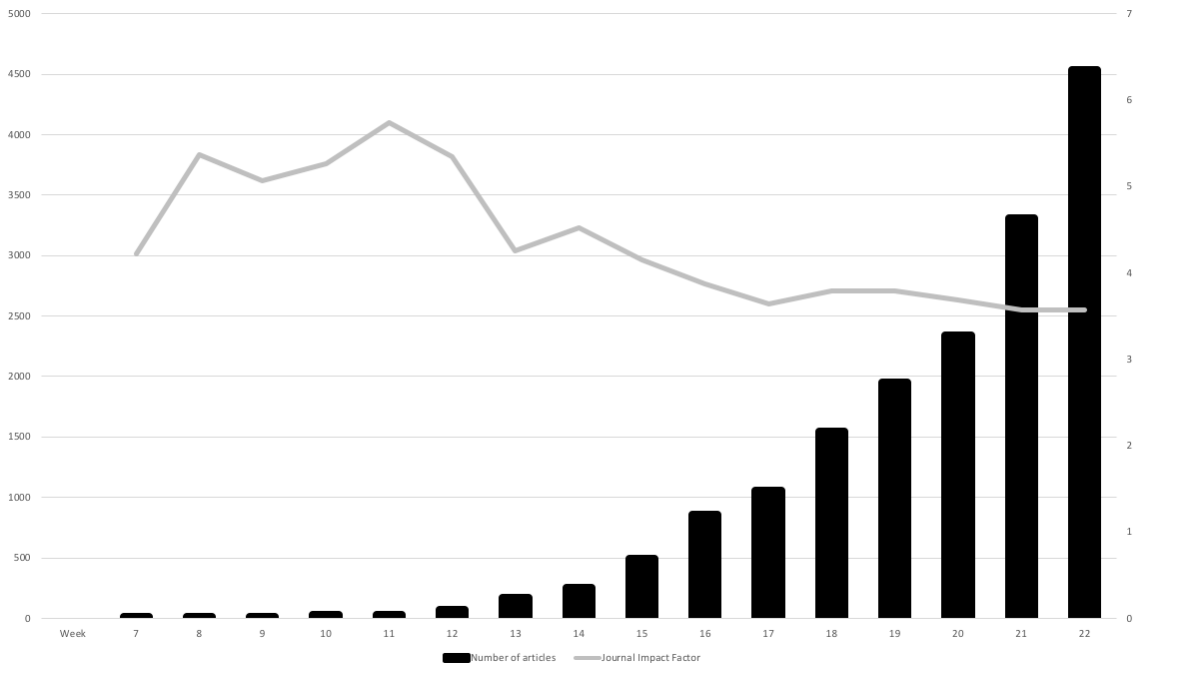
Number of articles published per week and the corresponding median journal impact factor.

The median weekly number of articles published was 374.5 (IQR 29.0-1648.5), ranging from 2 during week 7 (February 10-16, 2020) to 4536 during week 22 (May 25-30, 2020). The number of articles published per week shows an exponential development over time (Figure 2). The number of published case reports and review articles started to increase 8 and 11 weeks, respectively, after the first article was published. The number of published reports from randomized clinical trials or protocols for randomized clinical trials were uniformly spread throughout the study period. We conducted a manual search of the 23 articles containing either “randomiz*” or “randomis*” in the title and found that 6 (26%) of those were actual reports from randomized clinical trials.

### Analysis Based on LDA Modeling and Topics

**Figure 3:**
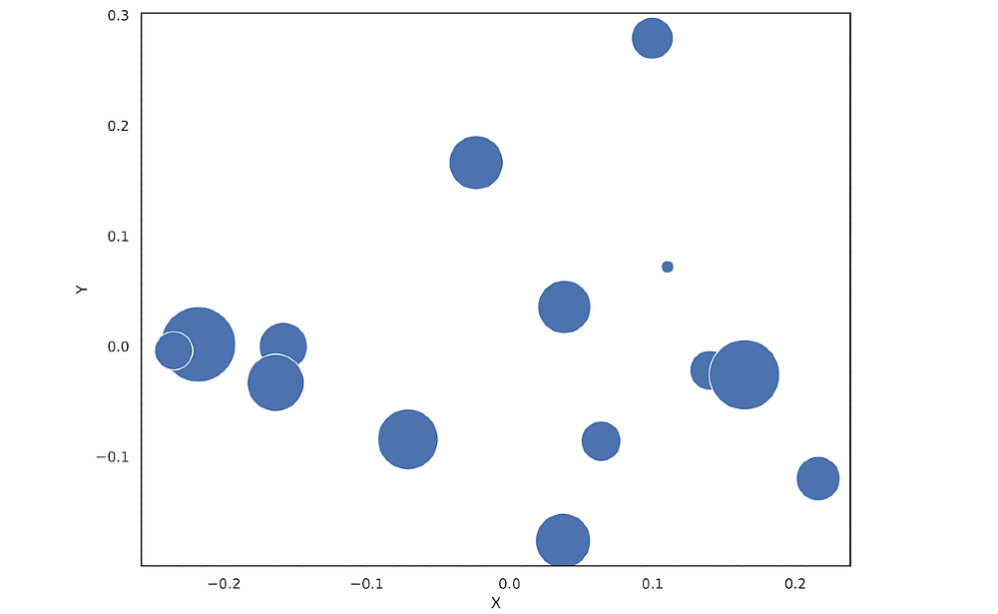
Principal component analysis plot for the chosen latent Dirichlet allocation model with 14 topics. Overlaps are seen for three topic clusters; however, these topics were found to be separated by clinical relevance.

The evaluation metrics perplexity and leave-out likelihood for LDA models with 3-23 topics (ie, the numbers of topics chosen based on a combination of expert opinion and arbitrary selection of 3 topics) exhibited decreasing and increasing values through the whole set; hence, these metrics provided little additional value with regard to choosing the optimal number of topics (Multimedia Appendix 3). In contrast, by assessing the PCA plots for all 20 models, we found the optimal number of topics (ie, the number of solutions with the least amount of overlapping topics) to be 14 (Figure 3). We found a low correlation between topic occurrence in the same article, indicating articles had well-defined topics (Multimedia Appendix 4).

The Pearson correlation coefficients ranged from 0.00 to 0.17, where the correlation between the topics health care response and clinical manifestations was the highest. Table 1 shows the labels, corresponding 5 most-frequent PubMed keywords and terms based on LDA, and the number of articles published for each topic. The main topics were health care response, with 2812 (16.86%) publications; clinical manifestations, with 1828 (10.96%) publications; and psychological impact, with 1771 (10.62%) publications. The least common topics were risk factors, laboratory diagnostics, and immunology.

**Table 1:** COVID-19 topics from latent Dirichlet allocation modeling.

Topic No.	Label	Five most-frequent terms based on latent Dirichlet allocation	Five most-frequent PubMed keywords	Journal impact factor, median (IQR)	Articles published, n (%) (N=16,670)
1	Laboratory diagnostics	antibody, time, laboratory, diagnostic, assay	pcr, rt, testing, disease, test	3.36 (2.0-6.1)	599 (3.59)
2	Therapies and vaccines	chloroquine, anti, hydroxychloroquine, pandemic, potential	hydroxychloroquine, chloroquine, drug, disease, antiviral	4.10 (2.9-6.6)	1193 (7.15)
3	Risk factors	ecmo, renin, respiratory, clinical, risk	diabetes, angiotensin, ace, disease, enzyme	4.13 (2.9-6.5)	420 (2.51)
4	Health care response	worker, response, practice, service, recommendation	health, pandemic, public, infection, disease	3.39 (2.4-5.1)	2812 (16.86)
5	Epidemiology	risk, control, datum, period, rate	disease, respiratory, epidemiology, novel, infection	4.09 (2.8-6.3)	819 (4.91)
6	Disease transmission	cause, spread, health, transmission, outbreak	respiratory, disease, syndrome, acute, virus	3.36 (2.5-6.2)	1141 (6.84)
7	Impact on health care practices	change, resident, time, virtual, visit	education, telemedicine, pandemic, health, medical	3.86 (2.5-5.7)	1115 (6.68)
8	Radiology	imaging, tomography, lesion, diagnosis, feature	pneumonia, tomography, computed, disease, ct	3.69 (2.7-5.5)	774 (4.64)
9	Epidemiological modeling	control, spread, measure, public, italy	health, pandemic, model, disease, public	3.48 (2.5-5.2)	1219 (7.31)
10	Clinical manifestations	increase, associate, infection, cardiovascular, injury	disease, acute, syndrome, respiratory, severe	4.99 (3.3-7.8)	1828 (10.96)
11	Protective measures	equipment, high, practice, perform, protective	surgery, cancer, pandemic, management, personal	4.50 (2.6-5.5)	1466 (8.79)
12	Immunology	expression, target, inhibitor, enzyme, viral	ace, angiotensin, protein, molecular, converting	4.56 (3.1-8.1)	694 (4.16)
13	Pregnancy	systematic, datum, include, disease, search	pregnancy, infection, respiratory, transmission, disease	3.52 (2.3-5.1)	819 (4.91)
14	Psychological impact	increase, stress, old, physical, public	health, pandemic, mental, social, anxiety	3.35 (2.4-5.0)	1771 (10.62)

**Figure 4:**
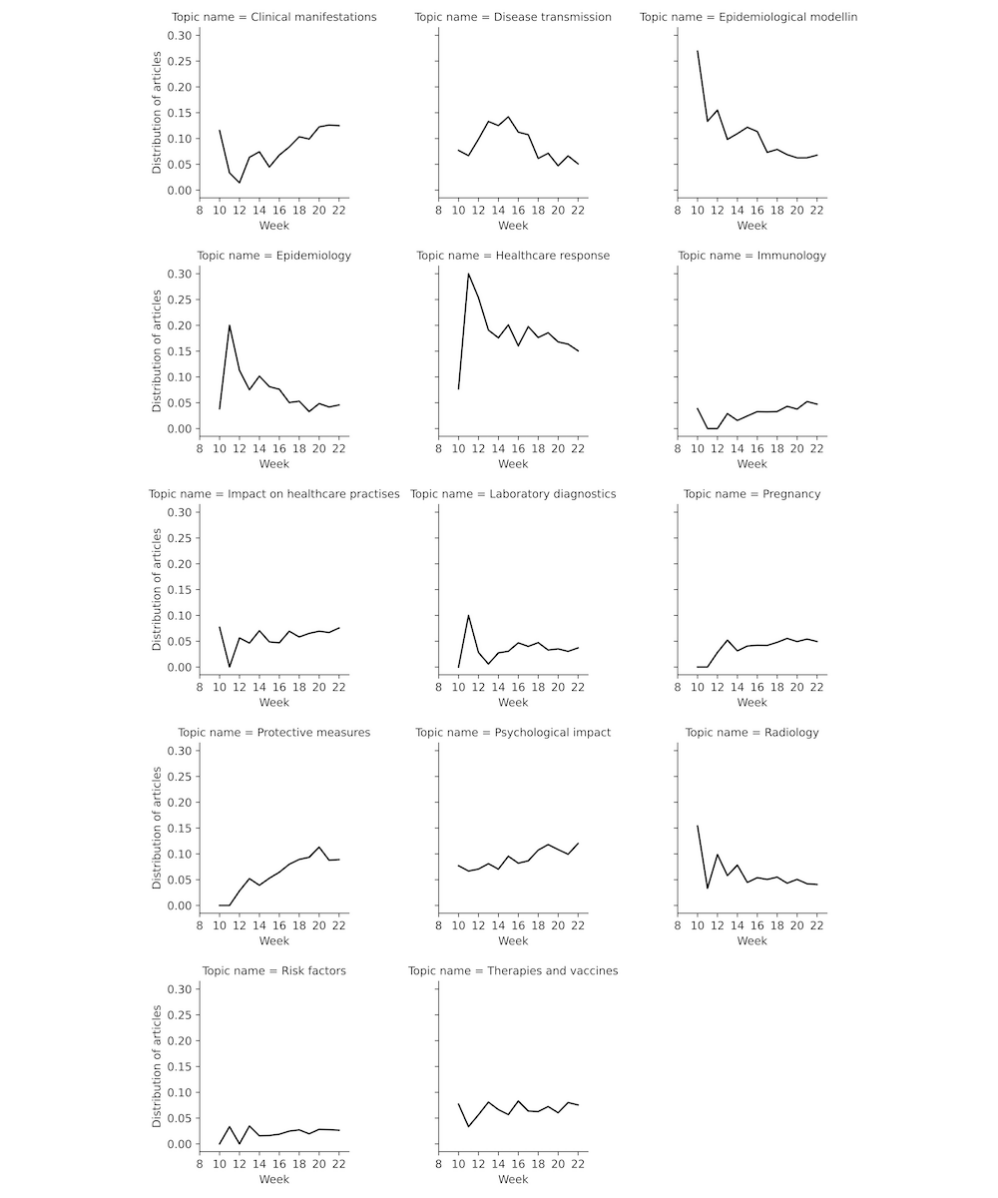
Proportion of topics in relation to all COVID-19 articles published per week.

Figure 4 shows the topic distribution over time. In particular, 4 topics (epidemiology, epidemiological modeling, health care response, and radiology) showed a declining curve over time, whereas 2 topics (clinical manifestations and protective measures) showed a clear increase in proportions, and 1 topic (disease transmission) showed a bell-shaped progression.

### Analysis Based on Journal Impact Factor

In all, the data set comprised 2473 unique journal names. Of these, we found perfect matches for 1129 (45.65%) journal names upon comparison with the Clarivate JIF list. For the remaining 1344 (54.34%) journal names, we used the random forest regression model to compute the JIF. The model showed an RMSE of 5.4 on test data (R^2^=47%), indicating that the average prediction erred with 5.4 points (Multimedia Appendix 5).

The median JIF for all articles in the data set was 3.7 (IQR 2.6-5.9). We found a declining trend in median JIF over time (Figure 2). The 3 topics with the highest median JIF were protective measures, immunology, and clinical manifestations (Table 1). There was a low correlation between the median JIF and the number of articles in each topic (Pearson correlation coefficient=−0.14).

### Analysis Based on Geographic Origin

**Figure 5:**
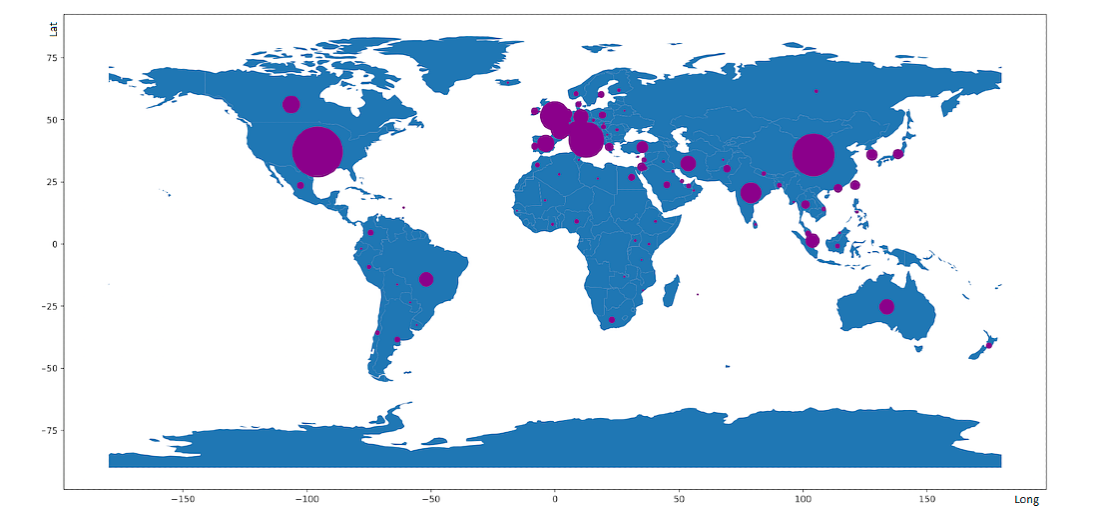
Geographic origins of the 16,670 published COVID-19 articles analyzed. Lat: latitude, Long: longitude.

Figure 5 shows the geographic origin of the 16,670 identified articles. The top 4 countries of origin were the United States (3223, 19.33%), China (2264, 13.58%), Italy (1591, 9.54%), and the United Kingdom (1055, 6.33%).

## Discussion

We classified the scientific publications on COVID-19 during the early phase of the pandemic into 14 topics. Overall, the most-prevalent topics were health care response, clinical manifestations, and psychological impact. Although the prevalence for some topics, such as health care response, has decreased over time, the prevalence for some other topics, such as clinical manifestations and protective measures, continues to increase. These findings suggest how research priorities have changed over time and, consequently, the topics that researchers consider relevant to study and publish have varied during the course of the pandemic. The 3 topics with the highest median JIF were risk factors, immunology, and clinical manifestations. Clinical manifestation was the only topic that featured in all of the following classifications: (1) the most prevalent topics, (2) the topics with the highest median JIF, and (3) the topics with an increasing prevalence. This finding indicates the importance of research on clinical manifestation during the early course of a pandemic caused by a novel virus. In addition, the results of this study show that the countries responsible for the most scientific outputs were also among the countries worst affected by the COVID-19 pandemic [20].

Moreover, we made a noteworthy finding that, in less than 6 months from the detection of the novel coronavirus, 6 peer-reviewed randomized trials on COVID-19 patients were published. The focus of these trials span from herbal [21] and medical [22-25] treatment options to respiratory rehabilitation [26]. It should be noted that such in-depth analysis of the data is not achieved by the topic model itself but requires some manual control.

When we did a comparative search for scientific articles published during the early phase of the 2009 influenza A (H1N1) pandemic [27], we made a strikingly different observation: The scientific community had a slower reaction to the H1N1 pandemic than to the COVID-19 pandemic, based on the date of publication of the first relevant scientific article, the overall number of publications, and the calculated median JIF. Moreover, the first randomized trial on H1N1 [28] was published (December 17, 2009) 190 days after the outbreak was declared as a pandemic; the corresponding timeframe for the first randomized trial on COVID-19 [23] was 44 days. This comparison, however, may be affected by many fundamental differences between the two pandemics, such as disease novelty and severity.

Previous studies have demonstrated the utility of topic modeling to map online activities [29,30], social media postings [31,32], and media reports on the COVID-19 pandemic [33]. To our knowledge, this is the first study to use topic modeling to assess published research on COVID-19. This study provides an in-depth analysis of a defined short period of time following the emergence of a novel disease. We believe our study findings may serve as an illustration of how the medical research community reacts, what topics are considered to be the most imperative to clarify, how research efforts are distributed geographically, and how they develop over time. Our analysis may also serve to demonstrate how research is being published, for instance, with regard to JIF when a new disease is discovered. Topic modeling enables an assessment of the research evolution, both on short and long term. The method could prove to be suitable for broad fields as well as narrow research questions. Topic modeling may also offer utility for additional in-depth analysis, by further exploring a selected topic to identify and analyze subtopics. Although several systematic reviews on COVID-19 have been published, it should be noted that such reviews do not feature the most recent literature; they are highly time- and resource-consuming; and they generally only focus on a specific aspect of the pandemic [10-12]. Using topic modeling, our study offers a comprehensive assessment of the available scientific publications on COVID-19. 

Our study has some limitations. First, the LDA model does not account for the context of a word, and a certain word may have different meanings depending on the context it is presented in. However, a review of our topics shows that this limitation was not prominent in our data. Therefore, it is unlikely that this limitation of the model negatively affects the interpretation of our topics. Second, there are several quantitative metrics to assess the optimal number of topics, which may conflict with the opinion of a subject matter expert. Any topic model produced, however, should be validated by subject matter experts, because any application of these topics will be done by such experts. Third, an article may appear in several topics in our data set. However, this may be considered a strength, as some overlap is indeed a property of research articles, and the aim of this study was to analyze scientific text in its original form. Fourth, the number of topics could be questioned—fewer topics may be easier for a reader to consume, whereas a larger number of topics could have resulted in a more mathematically optimal solution. However, we believe that a reasonable number of topics needs to be selected to balance mathematical accuracy and utility. Fifth, we limited our analyses to scientific publications. Analyses of grey literature may prove to add important information. Finally, as the COVID-19 pandemic is still ongoing, we expect the research topics to continuously change. To facilitate updated assessments, we have developed a web-based tool using the methods described in this study. Regular updates on the evolution of the COVID-19 evidence base can be found online at the c19research website [34].

Our study findings suggest that the scientific publications during the early phase of the COVID-19 pandemic can be modeled into topics. The evolution of these topics gives insights into current research trends and may aid researchers and policy makers to form a structured view of the existing COVID-19 evidence base and provide further research directions. Furthermore, our findings demonstrate that topic modeling is a rapid and useful method to assess the development of a broad and rapidly evolving research topic, such as COVID-19, and that it could be further utilized during the course of the current as well as future pandemics.

## Appendix

Multimedia Appendix 1 Stopwords used in the preprocessing of text data.

Multimedia Appendix 2 Top 15 characterizing words for each topic, from the final latent Dirichlet allocation model.

Multimedia Appendix 3 Evaluation metrics perplexity and leave-out likelihood for 20 latent Dirichlet allocation models.

Multimedia Appendix 4 Heatmap showing correlation between topic occurrence in the same article.

Multimedia Appendix 5 Histogram showing the distribution of journal impact factor (JIF) for the identified articles.

## Abbreviations

JIF: journal impact factor
LDA: latent Dirichlet allocation
PCA: principal component analysis
RMSE: root mean square error
