# Supplementary material for: Analysis of Scientific Publications During the Early Phase of the COVID-19 Pandemic: Topic Modeling Study
Source: J Med Internet Res. 2020 Nov 10;22(11):e21559. doi: 10.2196/21559 (PMC7674137; doi:10.2196/21559)
Supplement: Multimedia Appendix 1 [file jmir_v22i11e21559_app1.pdf]

'shall', 'accordance', 'yourself', 'successfully', 'each', 'showed', 'ok', 'eighty', 'welcome', 'their', ' those', 'been', 'whoever', 'ask', 'mean', 'thank', 'apparently', ' almost', 'cov', 'obtain', 'theyd', 'becomes', 'largely', 'date', 'except', 'n', 'herself', 'be', 'out', 'usefulness', 'itself', 'none', 'for', 'plus', 'went', 'coronavirus', 'your', 'studies', 'somewhere', 'make', 'give', 'means', 'etc', 'look', 'hereafter', 'nine', ' would', 'an', 'first', 'selves', 'vol', 'kept', 'im', 'yes', ' due', "shouldn't", 'awfully', 'mainly', 'therein', 'affects', 'patient', 'section', 'whereas', 'his', ' make', 'other', ' do', 'unto', 'probably', "doesn't", ' ml', 'together', 'fifth', 'some', 'when', 'anywhere', 'beginning', 'werent', ' therefore', 'value', ' that', 'previously', 'rather', 'ord', 'might', ' mg', 'due', ' what', 'home', 'he', 'found', 'after', 'its', 'et-al', 'na', 'ran', 'run', 'similar', 'readily', 'rd', 'over', 'whomever', ' several', 'a', 'thats', 'moreover', 'mg', 'myself', 'least', 'miss', 'r', 'thereupon', 'care', 'begins', 'sup', ' used', 'during', ' show', ' really', 'last', 'd', 'able', 'outside', 'fix', 'results', 'that', 'if', 'thereto', 'covid', 'behind', 'by', ' mainly', 'h', 'certainly', ' they', 'several', 'causes', 'mrs', 'qv', 'noone', 'say', 'shed', ' pmid', 'kg', 'following', 'different', 'thence', ' using', 'four', 'amongst', "it'll", 'otherwise', 'y', 'did', 'hereupon', 'themselves', 'nd', ' done', 'into', ' its', 'ex', 'himself', 'zero', 'see', 'ah', "you've", 'and', 'next', 'thanx', 'both', 'anybody', 'would', 'anyways', 'too', 'age', 'still', 'mug', 'patients', 'less', 'actually', 'everyone', 'regards', "they'll", 'name', 'oh', 'until', 'usually', 'afterwards', 'hid', 'one', 'more', 'specify', 'beyond', 'shows', 'whole', 'omitted', 'known', 'try', 'especially', 'itd', 'suggest', ' must', 'km', 'study', 'z', ' theirs', 'already', 'between', 'to', 'ie', 'yourselves', 'quite', 'thus', 'gone', 'think', 'whatever', 'you', 'up', ' seen', 'm', ' any', 'th', ' always', 'irr', ' etc', 'anyone', 'invention', 'past', 'suret', 'right', 'we', 'were', 'related', ' but', ' there', 'gotten', ' our', "there'll", 'sufficiently', 'various', 'obviously', 'ff', 'former', 'poorly', ' further', ' been', 'useful', 'besides', 'said', 'seemed', ' it', "don't", 'pp', "you'll", 'others', 'makes', 'hundred', 'because', 'nearly', "haven't", 'l', 'viz', 'lets', 'seeming', 'present', ' overall', 'important', 'somewhat', ' most', 'whod', 'slightly', 'ups', 'nobody', 'possibly', 'significant', 'at', 'brief', 'somehow', 'while', 'o', 'ed', 'whether', 'can', 'was', 'what', 'abst', 'everybody', 'ci', 'with', 'resulting', 're', 'cause', 'hes', 'quickly', 'unlike', 'namely', 'contain', "hasn't", 'should', 'ending', 'just', 'doing', 'mr', 'where', 'part', 'everywhere', 'corona', 'my', ' neither', 'much', 'obtained', ' we', 'sorry', ' another', 'use', 'coronaviruse', 'ninety', ' again', 'heres', 'per', 'theres', 'herein', ' during', 'become', 'seem', 'they', 'now', "that've", 'words', 'on', 'particular', 'are', 'away', 'such', 'but', 'indeed', ' nor', 'whereafter', 'ought', 'possible', 'wont', 'believe', 'elsewhere', 'here', 'which', 'trying', 'hardly', 'once', 'couldnt', ' their', 'similarly', 'particularly', 'nevertheless', "there've", 'comes', 'even', 'immediate', 'affecting', 'non', ' when', ' without', 'cannot', 'else', 'seen', 'then', 'throug', 'nor', 'under', 'although', 'or', 'stop', 'thanks', 'must', 'enough', 'old', 'sec', 'taking', 'having', 'she', 'us', 'maybe', 'co', 'importance', 'upon', 'against', 'further', 'neither', 'nos', 'nothing', 'not', 'in', 'little', "i'll", 'whither', 'thou', 'has', 'adj', 'either', ' use', 'v', 'regarding', 'respectively', 'please', ' could', 'gave', 'page', 'alone', 'seven', 'seems', 'mostly', ' itself', 'whereupon', 'unlikely', 'shes', ' especially', 'hereby', 'j', 'sars', 'wasnt', 'along', 'forth', 'take', 'x', 'come', 'sometime', 'all', 'really', 'everything', 'substantially', 'never', 'as', ' shows', 'since', 'beforehand', 'u', 'towards', 'usefully', ' because', 'til', "they've", 'sometimes', 'beginnings', 'whence', ' is', ' on', 'okay', 'edu', 'me', 'knows', 'willing', 'used', 'happens', 'median', 'i', 'pages', "we'll", 'around', 'own', 'un', 'than', 'uses', 'owing', 'thereby', 'end', 'says', 'vs', 'biol', 'who', 'becoming', 'way', ' being', 'without', 'wherein', 'getting', ' often', ' if', 'seeing', ' regarding', ' while', 'inward', 'got', 'specifying', 'down', 'predominantly', 'almost', 'anymore', 'nowhere', 'com', 'provides', 'whereby', 'two', 'every', 'ref', 'q', 'ones', 'liked', 'any', 'above', 'tries', 'sent', 'again', 'year', ' how', 'et', 'que', 'am', 'instead', 'new', 'nay', "that'll", ' since', 'later', 'merely', 'saying', ' of', 'lest', ' are', 'may', 'is', 'specifically', 'youd', ' should', 'proud', 'twice', 'tends', 'hospital', ' from', 'eg', ' into', 'using', 'made', ' the', 'get', 'e', 'needs', ' to', 'characteristic', 'ml', 'act', 'wheres', 'briefly', ' by', 'recent', 'latterly', 'effect', 'beside', ' although', 'her', 'www', 'know', 'need', 'announce', 'it', ' through', 'overall', 'million', "what'll", 'far', 'have', ' which', 'line', 'gets', 'normally', 'affected', 'w', 'k', ' all', 'hence', ' have', 'very', 'vols', 'wish', 'f', ' among', 'thereafter', 'taken', 'became', 'accordingly', 'begin', 'back', ' about', 'immediately', 'about', 'anyway', ' km', 'how', 'many', 'only', ' were', 'followed', 'no', "didn't", 'off', ' no', 'ca', 'few', 'show', "we've", 'whim', 'ltd', 'asking', 'noted', 'ourselves', ' significantly', ' mostly', 'whose', 'approximately', 'does', ' has', 's', 'sub', 'being', 'thoughh', 'strongly', ' thus', 'had', 'thru', 'nonetheless', ' within', 'aren', 'truly', 'meanwhile', 'whenever', 'necessary', ' shown', 'according', 'specified', 'hither', 'could', 'arent', 'want', 'of', 'looking', 'goes', 'unfortunately', 'across', 'index', 'available', 'tell', ' mm', 'id', 'however', 'gives', 'hers', 'so', 'g', 'resulted', 'throughout', 'p', 'from', 'done', "isn't", 'let', 'ours', 'do', ' then', ' might', 'go', 'primarily', ' in', "can't", 'those', 'six', 'within', 'him', 'day', ' may', 'before', ' them', 'necessarily', 'wants', ' also', 'our', 'theyre', 'containing', 'auth', 'yet', 'regardless', ' p', 'somethan', 'given', 'five', 'information', "'ll", 'toward', 'there', ' having', "i've", 'like', ' so', 'them', 'took', ' very', 'same', 'the', 'most', 'always', 'keepkeeps', 'promptly', 'this', 'hi', ' an', 'significantly', 'research', 'put', 'anything', 'thousand', 'potentially', 'shown', 'ts', "'ve", 'relatively', 'howbeit', 'perhaps', ' between', 'tip', ' such', 'thereof', ' enough', ' here', 'theirs', 'often', 'also', 'self', 'though', 'wouldnt', ' does', 'eight', 'refs', 'ncov', ' before', 'likely', 'unless', ' showed', 'thered', 'aside', ' either', 'whom', ' these', 'came', 'ever', "who'll", 'anyhow', 'onto', 'among', 'formerly', 'certain', 'hed', 'latter', 'looks', 'via', 'inc', 'downwards', 'near', 'below', 'therere', 'youre', 'yours', ' however', 'whos', 'b', 'something', ' and', 'added', 'another', 'lately', "she'll", 'tried', 'wed', ' found', 'giving', 'arise', 'someone', 'these', 'widely', ' with', 'recently', 'meantime', 'furthermore', 'why', 'c', 'follows', 'soon', 'placed', 'somebody', ' both', 'showns', 'whats', 'wherever', 'saw', 'world', 'contains', 'therefore', 'through'
