# Supplementary material for: Analysis of Scientific Publications During the Early Phase of the COVID-19 Pandemic: Topic Modeling Study
Source: J Med Internet Res. 2020 Nov 10;22(11):e21559. doi: 10.2196/21559 (PMC7674137; doi:10.2196/21559)
Supplement: Multimedia Appendix 2 [file jmir_v22i11e21559_app2.pdf]

Top 15 words for the topic Laboratory diagnostics
'antibody', 'time', 'laboratory', 'diagnostic', 'assay', 'rna', 'negative', 'testing', 'viral', 'detection', 'sample', 'rt', 'positive', 'pcr', 'test'

Top 15 words for the topic Therapies and vaccines
'chloroquine', 'anti', 'hydroxychloroquine', 'pandemic', 'potential', 'antiviral', 'infection', 'vaccine', 'therapeutic', 'therapy', 'trial', 'clinical', 'disease', 'drug', 'treatment'

Top 15 words for the topic Risk factors
'ecmo', 'renin', 'respiratory', 'clinical', 'risk', 'diabetes', 'therapy', 'convalescent', 'severe', 'disease', 'treatment', 'angiotensin', 'plasma', 'blood', 'diabete'

Top 15 words for the topic Healthcare response
'worker', 'response', 'practice', 'service', 'recommendation', 'resource', 'challenge', 'provide', 'medical', 'emergency', 'disease', 'management', 'healthcare', 'health', 'pandemic'

Top 15 words for the topic Epidemiology
'risk', 'control', 'datum', 'period', 'rate', 'child', 'transmission', 'symptom', 'confirm', 'report', 'contact', 'china', 'infection', 'disease', 'case'

Top 15 words for the topic Disease transmission
'cause', 'spread', 'health', 'transmission', 'outbreak', 'china', 'human', 'novel', 'severe', 'acute', 'infection', 'syndrome', 'disease', 'virus', 'respiratory'

Top 15 words for the topic Impact on healthcare practices
'change', 'resident', 'time', 'virtual', 'visit', 'online', 'practice', 'telehealth', 'program', 'technology', 'student', 'telemedicine', 'education', 'medical', 'pandemic']

Top 15 words for the topic Radiology
'imaging', 'tomography', 'lesion', 'diagnosis', 'feature', 'symptom', 'image', 'finding', 'case', 'disease', 'lung', 'chest', 'clinical', 'pneumonia', 'ct'

Top 15 words for the topic Epidemiological modelling
'control', 'spread', 'measure', 'public', 'italy', 'health', 'disease', 'china', 'datum', 'country', 'number', 'outbreak', 'case', 'epidemic', 'model'

Top 15 words for the topic Clinical manifestations
'increase', 'associate', 'infection', 'cardiovascular', 'injury', 'syndrome', 'outcome', 'mortality', 'high', 'clinical', 'respiratory', 'risk', 'acute', 'severe', 'disease'

Top 15 words for the topic Protective measures
'equipment', 'high', 'practice', 'perform', 'protective', 'surgeon', 'aerosol', 'treatment', 'management', 'procedure', 'risk', 'surgical', 'surgery', 'pandemic', 'cancer'

Top 15 words for the topic Immunology
'expression', 'target', 'inhibitor', 'enzyme', 'viral', 'response', 'virus', 'cytokine', 'infection', 'angiotensin', 'immune', 'receptor', 'protein', 'ace', 'cell'

Top 15 words for the topic Pregnancy
'systematic', 'datum', 'include', 'disease', 'search', 'pregnancy', 'evidence', 'pregnant', 'case', 'symptom', 'clinical', 'woman', 'report', 'review', 'infection'

Top 15 words for the topic Psychological impact
'increase', 'stress', 'old', 'physical', 'public', 'population', 'anxiety', 'psychological', 'people', 'risk', 'impact', 'social', 'mental', 'pandemic', 'health'
