# Supplementary figures and images for: Analysis of Scientific Publications During the Early Phase of the COVID-19 Pandemic: Topic Modeling Study
Source: J Med Internet Res. 2020 Nov 10;22(11):e21559. doi: 10.2196/21559 (PMC7674137; doi:10.2196/21559)

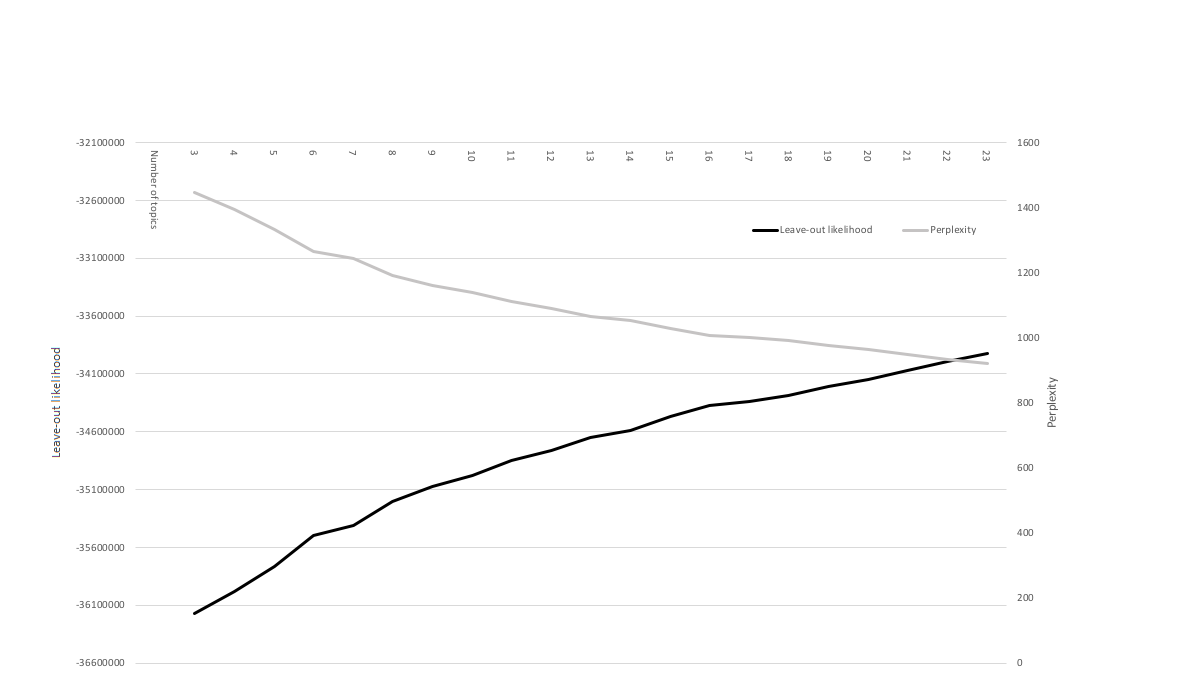

Supplement: Multimedia Appendix 3 [file jmir_v22i11e21559_app3.png]

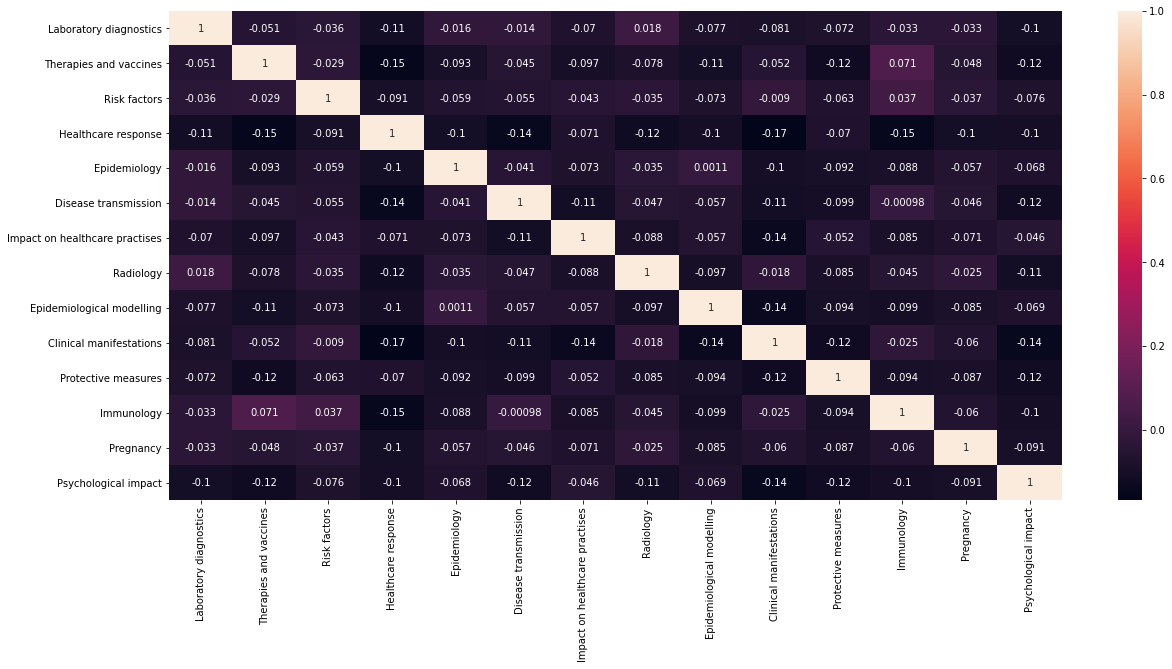

Supplement: Multimedia Appendix 4 [file jmir_v22i11e21559_app4.png]

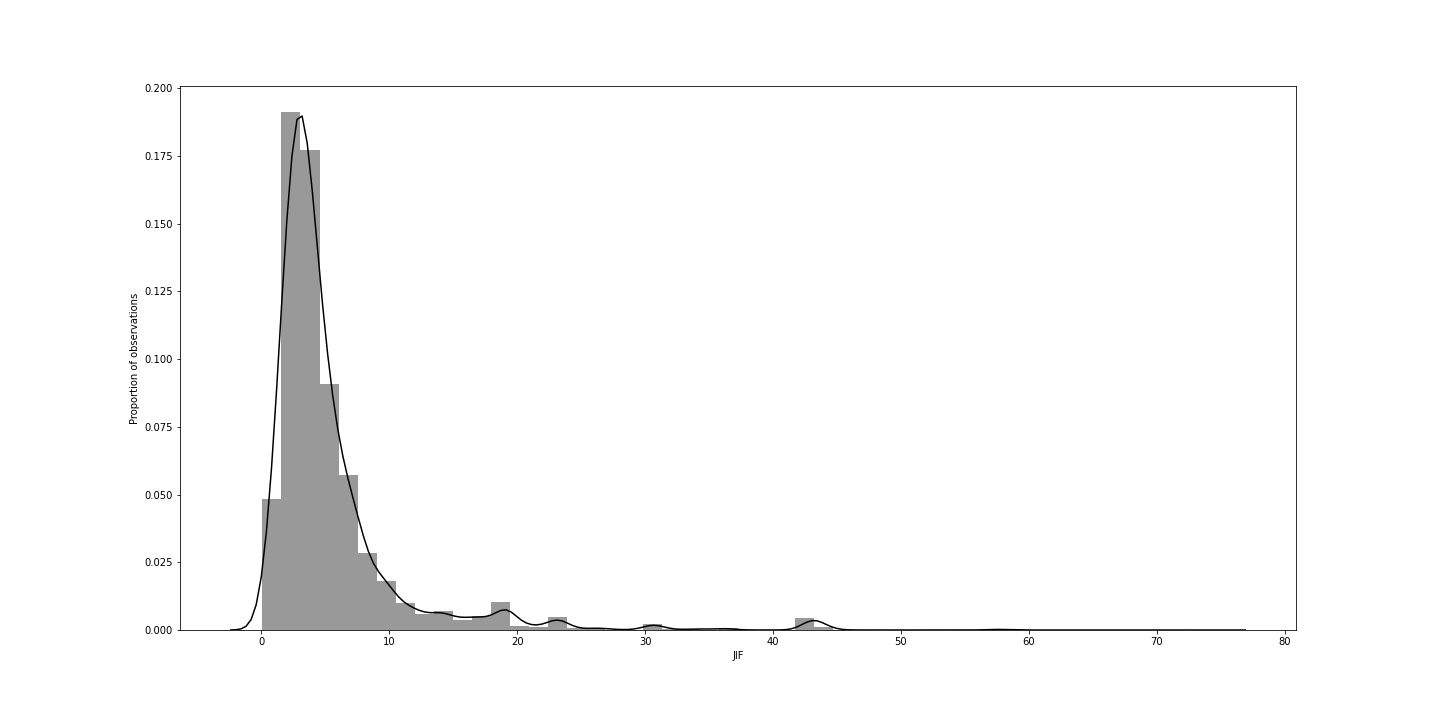

Supplement: Multimedia Appendix 5 [file jmir_v22i11e21559_app5.png]
